# Nivolumab-Induced Toxic Epidermal Necrolysis: Rare but Fatal Complication of Immune Checkpoint Inhibitor Therapy

**DOI:** 10.7759/cureus.15017

**Published:** 2021-05-13

**Authors:** Michael C Kim, Huda N Khan

**Affiliations:** 1 Department of Medicine, Graduate Medical Education, Methodist Dallas Medical Center, Dallas, USA; 2 Internal Medicine, Methodist Health System, Dallas, USA

**Keywords:** toxic epidermal necrolysis, nivolumab, immune checkpoint inhibitor, scorten, intravenous immunoglobulin

## Abstract

Toxic epidermal necrolysis (TEN) is a rare, but potentially fatal dermatological emergency most commonly caused by medication exposure. It is characterized by skin desquamation affecting over 30% of the body, and it remains a fatal condition with a high mortality rate. Nivolumab, an immune checkpoint inhibitor used in the treatment of various types of malignancies, has been linked to TEN. Nivolumab-induced TEN is a rare phenomenon with a low incidence rate in patients treated with a single-agent immune checkpoint inhibitor, but it has a high mortality rate that exceeds non-nivolumab-induced TEN. Nivolumab-induced TEN can present with many potential complications such as hemodynamic instability from excessive fluid loss, sepsis from bacterial superinfection, and disseminated intravascular coagulation. Due to its high mortality rate, prompt recognition of the condition, immediate withdrawal of the offending drug(s), vigorous skin care, multispecialty collaboration, and close monitoring of complications is needed. We present a case of nivolumab-induced TEN in an elderly male with a history of hepatocellular carcinoma who presented with acute-onset skin desquamation after nivolumab initiation.

## Introduction

Toxic epidermal necrolysis (TEN), also called Lyell syndrome, is a rare and potentially fatal dermatological emergency with an incidence of 0.4 to 1.9 cases per million annually and a mortality rate of 30% at one year [1,2]. It is characterized by diffuse erythema, bullous detachment, and necrosis of the epidermis and mucosal membranes, which leads to epidermal desquamation and possible death [1]. Despite advancement in the knowledge of TEN pathophysiology and management, it remains a fatal condition with a high mortality rate [1]. First described by Lyell in 1956, TEN has been associated with infections, but more than 80% of cases are drug-induced [1,2]. Nivolumab (Opdivo) is an immune checkpoint inhibitor used in various types of malignancies such as melanoma, hepatocellular carcinoma, colorectal cancer, Hodgkin’s lymphoma, and many more, with common side effects such as fatigue, cough, dyspnea, and rashes; however, it has also been documented to cause rare life-threatening conditions such as Stevens-Johnson syndrome (SJS) and TEN with a mortality rate above 70% [1-3]. Nivolumab-induced TEN is an extremely rare phenomenon and requires prompt recognition, immediate withdrawal of offending drug(s), vigorous skin care, multispecialty collaboration, and close monitoring of complications. We present a case of nivolumab-induced TEN in an elderly male with a history of hepatocellular carcinoma who presented with acute-onset skin desquamation after nivolumab initiation.

## Case presentation

An 86-year-old Vietnamese male with a past medical history of type 2 diabetes mellitus, hypothyroidism, Parkinson’s dementia, hepatitis C complicated by liver cirrhosis, and hepatocellular carcinoma presented with a two-week history of worsening skin rash. According to the patient, the rash appeared on his upper torso and progressed to involve the majority of his chest, back, and arms. A few days prior to presentation, the rash started to blister and burst, which was followed by skin denudation. He endorsed associated itching and pain. The patient had undergone two cycles of nivolumab therapy for the treatment of his hepatocellular carcinoma, with the last dose administered two weeks prior to the onset of the rash. His oncologist initially treated the rash with a course of prednisone (40 mg daily), but due to lack of improvement, he presented to the hospital. The patient denied fever, recent travel, illness, sore throat, chest pain, dyspnea, nausea, vomiting, diarrhea, dysuria, myalgia, joint swelling, or dizziness.

**Figure 1:**
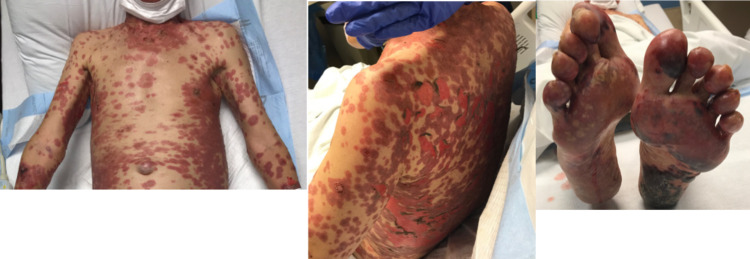
Initial presentation of nivolumab-induced toxic epidermal necrolysis.

On admission, the patient had a temperature of 36.6°C, a heart rate of 75 beats per minute, a respiratory rate of 20 breaths per minute, and a blood pressure of 155/72 mmHg. Physical examination was significant for lesions in various stages of evolution, including many bullae and three-ringed targetoid lesions on the anterior thorax, extensive skin sloughing, and denudation over the chest, abdomen, and bilateral feet (Figure 1). There was minimal superficial crusting on the lips, and initial sparing of intraoral mucosa, eyes, genitals, and anal mucosa. A laboratory panel showed a normal white blood cell count (7.6 × 10^3^/uL; reference range: 3.8-10.6 × 10^3^/uL), a low hemoglobin (12.0 g/dL; reference range: 13.5-17.5 g/dL), a low platelet count (8.7 × 10^3^/uL; reference range: 130-400 × 10^3^/uL), mildly elevated aspartate transaminase level (65 U/L; reference range: 8-42 U/L), and mildly elevated alanine aminotransferase level (58 U/L; reference range: <50 U/L).

Based on the patient’s clinical history and skin desquamation involving >30% of his total body surface, TEN was high on the differential diagnosis which included but was not limited to SJS, erythema multiforme, drug hypersensitivity syndrome, and bullous pemphigoid. The SCORTEN (Score of Toxic Epidermal Necrolysis) score was calculated to be 3, indicating a mortality rate of 35.5%. Therefore, the patient was admitted to the intensive care unit (ICU) for closer monitoring. Due to the extensive skin involvement and concern for fatal complications, a multidisciplinary team approach to management was taken, and dermatology, oncology, and wound care physicians were consulted.

**Figure 2:**
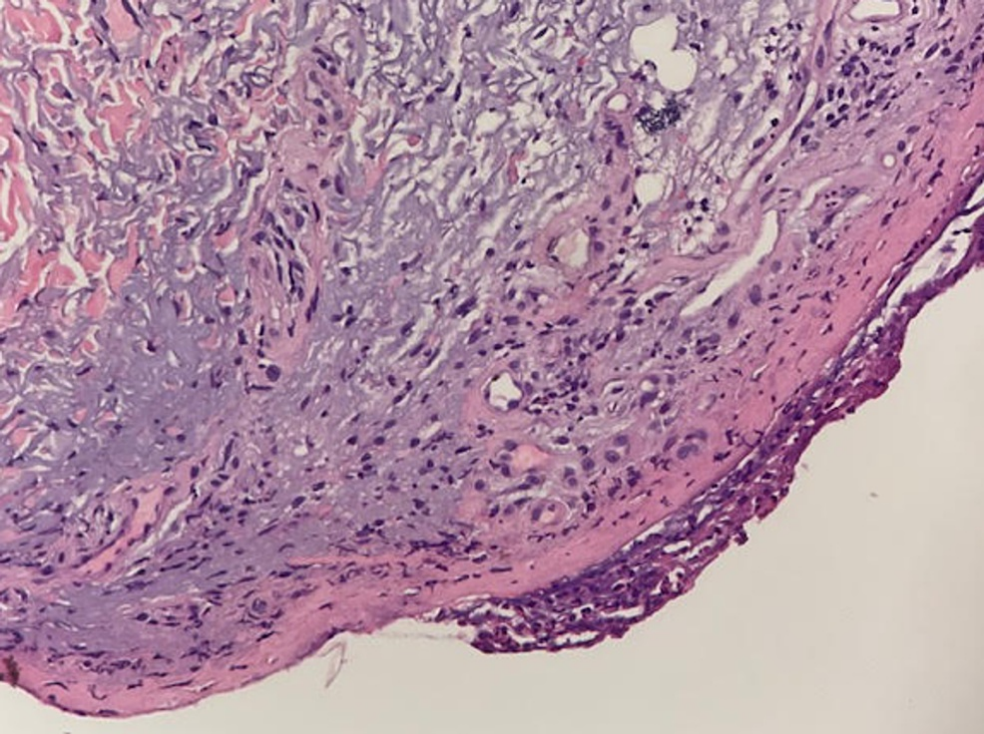
Skin biopsy showing epidermis with dyskeratotic cells with focal full-thickness necrosis of the roof and superficial dermis with sparse lymphocytic infiltrate and scattered erythrocytes (H&E stain). H&E: hematoxylin and eosin

A skin biopsy was performed which showed numerous dyskeratotic cells in the epidermis, focal full-thickness necrosis of the roof of an acute blister and superficial dermis, sparse lymphocytic infiltrate, and scattered erythrocytes (Figure 2). Immunohistochemistry did not detect IgG, IgA, IgM, and C3, indicating absence of an immunobullous disorder such as nivolumab-induced bullous pemphigoid. *Mycoplasma pneumoniae* IgM antibody was also not detected. Therefore, biopsy results together with the patient’s clinical presentation were consistent with a diagnosis of nivolumab-induced TEN. The patient was managed with intravenous fluids, pain control, and local wound care. According to dermatology and oncology recommendations, the patient was also treated with intravenous solumedrol (250 mg once daily for four days) and intravenous immunoglobulin (IVIG) (60 g of 10% infusion IVIG for five days).

**Figure 3:**
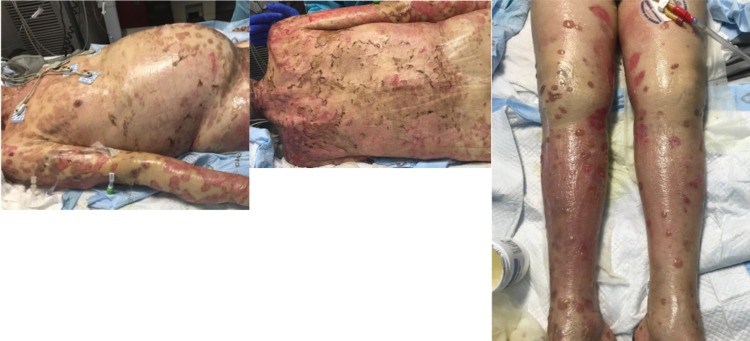
Hospitalization day 14, post intravenous immunoglobulin and high-dose steroid therapy.

While the patient’s cutaneous condition improved and re-epithelialization had begun to occur (Figure 3), his hospital course was complicated by polymicrobial sepsis. Urine culture was positive for *Klebsiella pneumoniae* and *Enterococcus faecalis*, and blood culture was positive for *Acinetobacter baumannii* and diphtheroids. Infectious diseases was consulted and the patient was started on empirical intravenous meropenem (500 mg every eight hours). The antibiotic regimen was later narrowed to intravenous ampicillin and sulbactam (1.5 g every six hours) based on culture sensitivities.

Unfortunately, the patient’s overall condition declined. He developed disseminated intravascular coagulation (DIC) likely due to the sepsis, TEN, and underlying malignancy. Laboratory values were significant for thrombocytopenia (31 × 10^3^/uL; reference range: 130-400 × 10^3^/uL), low fibrinogen (160 mg/dL; reference range: 214-481 mg/dL), elevated prothrombin time (19.9 seconds; reference range: 11.3-14.7 seconds), normal partial thromboplastin time (28 seconds; reference range: 23-37 seconds), elevated international normalized ratio (1.6; reference range: 0.9-1.2), and elevated D-dimer (13.81 ug/mL FEU; reference range: 0.00-0.50 ug/mL FEU). He was managed supportively with platelet and cryoprecipitate transfusions. After a goals-of-care discussion with the palliative care team, the patient’s family elected to change the patient’s code status to do-not-resuscitate. On day 20 of hospitalization, the patient began to rapidly deteriorate with the development of acute encephalopathy, respiratory distress, and worsening hypotension requiring pressor support, and the patient succumbed to death secondary to superinfection and DIC.

## Discussion

TEN is the most severe form of acute blistering disease, which also includes erythema multiforme, SJS, and overlap syndrome [1]. The blistering diseases differ in percentage of total body skin involvement. In erythema multiforme and SJS, skin involvement is <10% of total body surface; in overlap syndrome, skin involvement is 10% to 30%; and in TEN, skin involvement is >30% [1]. The combined incidence of SJS, overlap syndrome, and TEN is two to seven cases per million annually, whereas TEN alone has a reported incidence of 0.4 to 1.9 cases per million annually [1]. TEN can occur in patients of all ages, and it occurs more commonly in women, with a female-to-male ratio of 1:7. TEN frequently occurs in immunosuppressed states such as bone marrow transplant, malignancy, connective tissue disease, and human immunodeficiency virus [1,4].

TEN is believed to be caused by keratinocyte apoptosis due to an inappropriate immune response to certain drugs, infections, or vaccinations [1]. There are various theories behind the mechanism of TEN, but it is believed that cytotoxic CD8+ T lymphocytes and natural killer cells are the main inducers of keratinocyte apoptosis [1]. The two cells produce Fas ligand (FasL), which binds to Fas on target cells, subsequently activating the Fas/FasL-associated signaling pathway that induces apoptosis [5]. The CD8+ T lymphocytes and natural killer cells also produce the cytolytic peptide granulysin, further contributing to apoptosis [5].

Nivolumab is believed to cause TEN via inhibition of programmed death receptor-1 (PD-1) [6]. PD-1 is an immune inhibitory checkpoint receptor found on activated T cells. When PD-1 interacts with its ligands PD-L1 and PD-L2, T cell function is exhausted [6]. Nivolumab blocks the interaction between PD-1 and its ligands, which leads to prolonged T cell responses for cancer treatment [6]. However, due to unknown reasons, the lasting T cell response can lead to autoimmune or inflammatory response in normal tissue resulting in apoptosis via the mechanism mentioned above.

As over 80% of TEN cases are triggered by drug exposure, a thorough medication reconciliation must be done [2]. Numerous drugs such as anticonvulsants (e.g., lamotrigine, carbamazepine, phenytoin, and phenobarbital), sulfonamide antibiotics, allopurinol, non-steroidal anti-inflammatory drugs, and immune checkpoint inhibitors (e.g., nivolumab) have been linked to TEN, so recognition and immediate withdrawal of the suspected agent is crucial [1,5]. Nivolumab-induced TEN typically occurs within one month of treatment initiation [6]. While low-grade skin reactions such as pruritis, vitiligo, and mucositis occur in 17% to 40% of patients treated with nivolumab, nivolumab-induced TEN is a rare phenomenon that occurs in only 2% to 3% of patients treated with a single-agent immune checkpoint inhibitor [3,6]. Furthermore, while the overall mortality rate of TEN is about 30%, nivolumab-associated TEN has a grim prognosis with a mortality rate above 70% [2,3].

TEN is largely a clinical diagnosis as there are no specific diagnostic criteria or histological findings. Physicians must have a complete understanding of the patient’s clinical history, the context, and potential risk factors that may contribute to the development of TEN [4]. The cutaneous manifestation of TEN is generally preceded by fever (>39°C), cough, conjunctivitis, or malaise. Symmetrical and painful macular exanthem subsequently develops on the face and trunk before spreading to the extremities [5]. Patients develop Nikolsky sign, where gentle lateral traction of the skin causes the epidermis to slough off, exposing the dermis [5]. In 90% of TEN cases, there is mucosal involvement in the oral, ocular, or urogenital regions [4]. Desquamation of the skin can progress up to seven to twelve days and re-epithelialization occurs anywhere from one to three weeks [1,5]. On the other hand, mucosal denudation involving areas such as gastrointestinal, respiratory, or genitourinary tract may take months before complete recovery [5].

When evaluating TEN, physicians must obtain laboratory studies, including complete blood count, chemistry panel, and albumin level as TEN patients often exhibit electrolyte imbalance and hypoalbuminemia due to a hypercatabolic state and severe transdermal fluid loss [4]. Patients suffering from TEN have a high risk of sepsis from bacterial superinfection, so blood cultures should also be obtained [4]. A SCORTEN score must also be calculated to determine the acuity and level of care the patient may require as higher SCORTEN scores are correlated with higher mortality (Table 1) [1,5]. This was evidenced by the patient in our case who was admitted to the ICU for close monitoring because of his high SCORTEN score and high mortality risk.

**Table 1:** Toxic epidermal necrolysis severity-of-illness (SCORTEN) score*. *Total score (mortality rate) (adapted from [7]): 0-1 (3.2%); 2 (12.2%); 3 (35.5%); 4 (58%, 3%); >5 (90.0%)

Criteria	Score
Age >40 years	1
Heart rate >120 beats per minute	1
Diagnosis of malignancy	1
Epidermal detachment >10% of body surface on day one of hospitalization	1
Blood urea nitrogen >28 mg/dL	1
Glucose >252 mEq/L	1
Bicarbonate <20 mEq/L	1
SCORTEN	7

Furthermore, although only a small number of TEN cases have been reported to be caused by measles-mumps-rubella vaccination and *Mycoplasma pneumoniae* infection, vaccination history must be obtained and infectious etiologies must be ruled out during TEN evaluation [1]. Skin biopsy is generally recommended with an appropriate sample size being a large >4 mm punch biopsy or a deep shave biopsy [4]. In TEN, skin biopsy shows confluent necrosis of cells in the epidermis, keratinocyte apoptosis, dermo-epidermal junction separation, and T lymphocyte infiltrates in the dermis [1,4,5]. As these findings are not specific or diagnostic for TEN, clinical correlation with the patient’s history and risk factors must be weighed to support the diagnosis of TEN [4].

**Figure 4:**
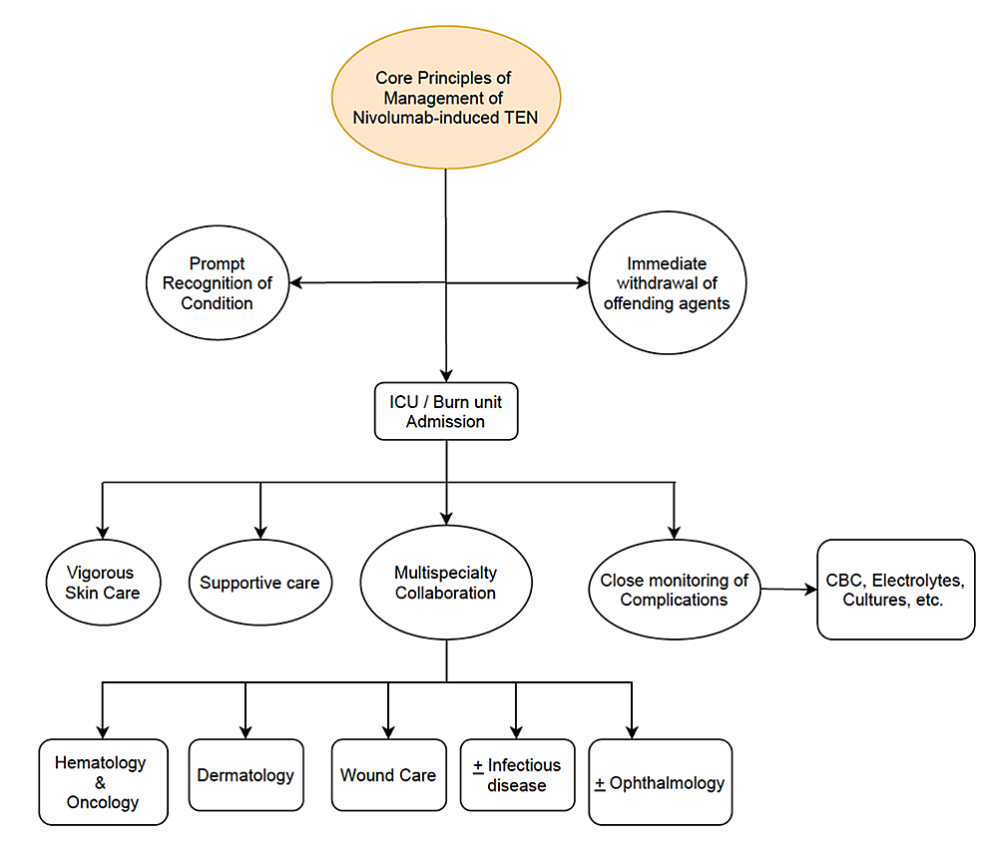
Core principles of the management of nivolumab-induced toxic epidermal necrolysis. CBC: complete blood count; ICU: intensive care unit; TEN: toxic epidermal necrolysis

Because there is no definitive cure for TEN, multispecialty collaboration involving various specialties such as Wound Care, Dermatology, Ophthalmology, Hematology/Oncology, and Infectious Diseases is crucial in the management of TEN and its potential complications (Figure 4). As with any patient with extensive skin loss, the most common cause of hemodynamic complications and potential hypovolemic shock is from excessive fluid loss, so wound care and intravenous fluid resuscitation are critical in patient care [1]. Desquamated skin also poses an increased risk for sepsis from bacterial superinfection and multiorgan failure; therefore, close monitoring for signs of infection is also required [1]. Ophthalmological complications occur in 50% to 90% of patients with TEN, so if there is any sign or suspicion for ocular involvement, Ophthalmology should be consulted [1]. A collaborative effort was made in managing the patient in our case, and wound care and intravenous fluid resuscitation were at the forefront of management.

Over the years, various treatments such as steroids, cyclosporine, cyclophosphamide, plasmapheresis, and IVIG have been proposed for TEN. However, due to lack of sufficient evidence for definitive treatment recommendation, treatment risks and benefits must be weighed, and Hematology/Oncology must be consulted prior to initiation of therapy [8]. As TEN is believed to be due to inappropriate immune responses, steroids are considered to be a first-line treatment for TEN [8]. Various doses of prednisone equivalents have shown a potential benefit in the treatment of TEN. Moreover, methylprednisolone pulse therapy has shown to decrease levels of pro-inflammatory cytokines, including TNF-α, IL-6, and IFN-γ [8]. However, studies have shown that steroid use can lead to possible bacterial superinfection and delayed wound healing. Therefore, definitive guidelines for steroid use in TEN are yet to be established [8].

Cyclosporine is another drug that can be used for TEN management [8]. Cyclosporine specifically targets granulysin, thus reducing an inappropriate immune response and keratinocyte apoptosis [8]. A small clinical trial from 2010 involving TEN patients who each received 3 mg/kg/day of cyclosporine for 10 days showed epidermal detachment stabilization [8]. Cyclophosphamide is another drug that has been tested in the past due to its ability to inhibit CD8 [8]. However, due to lack of studies and sufficient evidence, there are no specific guidelines for the use of cyclosporine or cyclophosphamide [5]. Further, plasmapheresis has been used in the past as adjunctive therapy with the goal of clearing cytokines and drug metabolites [5]. While small studies have shown its potential benefits, further studies are needed to determine its efficacy in TEN treatment [8].

As one of the most commonly used immunomodulators, IVIG has been used in the management of TEN [7]. Anti-Fas antibodies in IVIG putatively prevent apoptosis via inhibition of the Fas/FasL pathway [6]. Both low- and high-dose IVIGs (up to 2 g/kg) have been used with positive outcomes, and each 1 g/kg increase in IVIG dose correlated with a 4.2-fold increase in survival rate [8,9]. Furthermore, small studies in China and India have shown that combined IVIG and steroid therapies are more effective treatments with faster disease resolution and reduced mortality [8,10,11]. While these studies have shown a potential benefit of IVIG or combined steroid and IVIG therapy, further studies must be done to establish the most effective doses for IVIG and steroid treatment [5]. Also, IVIG has been known to increase the risk for aseptic meningitis and thromboembolic events, so risks and benefits must be weighed prior to initiation.

Lastly, complications of TEN such as bacterial superinfection and DIC must be closely monitored for and managed. Daily vital signs, physical examination, and complete blood count must be monitored for signs of infection. If there is any suspicion of infection, empiric antibiotic coverage for methicillin-resistant *Staphylococcus aureus* and *Pseudomonas aeruginosa* must be initiated until specific culture data from blood and urine cultures is obtained [4]. Also, while uncommon, signs of DIC must be monitored as it is associated with increased mortality [12]. SJS/TEN patients diagnosed with DIC had a greater than 10-fold increase in mortality compared to those not diagnosed with DIC (78.1% vs. 7%) [12]. Unfortunately, our patient succumbed to both superinfection and DIC, which ultimately lead to his demise.

## Conclusions

Nivolumab-induced TEN is a rare but fatal dermatological emergency. A number of cases have been reported over the years and numerous therapies have been trialed, but definitive treatment recommendations are yet to be defined and further studies are needed. Despite the lack of concrete guidelines, physicians can still provide optimal care through prompt recognition of the condition, immediate withdrawal of offending drug(s), vigorous skin care, multispecialty collaboration, and close monitoring of complications.
